# Natural Killer Cell Receptor Genes in the Family *Equidae*: Not only *Ly49*

**DOI:** 10.1371/journal.pone.0064736

**Published:** 2013-05-28

**Authors:** Jan Futas, Petr Horin

**Affiliations:** 1 Departmen of Animal Genetics, University of Veterinary and Pharmaceutical Sciences Brno, Brno, Czech Republic; 2 CEITEC-VFU, University of Veterinary and Pharmaceutical Sciences Brno, Brno, Czech Republic; INSERM- CNRS- Univ. Méditerranée, France

## Abstract

Natural killer (NK) cells have important functions in immunity. NK recognition in mammals can be mediated through killer cell immunoglobulin-like receptors (KIR) and/or killer cell lectin-like Ly49 receptors. Genes encoding highly variable NK cell receptors (NKR) represent rapidly evolving genomic regions. No single conservative model of NKR genes was observed in mammals. Single-copy low polymorphic NKR genes present in one mammalian species may expand into highly polymorphic multigene families in other species. In contrast to other non-rodent mammals, multiple *Ly49*-like genes appear to exist in the horse, while no functional *KIR* genes were observed in this species. In this study, *Ly49* and *KIR* were sought and their evolution was characterized in the entire family *Equidae.* Genomic sequences retrieved showed the presence of at least five highly conserved polymorphic *Ly49* genes in horses, asses and zebras. These findings confirmed that the expansion of *Ly49* occurred in the entire family. Several *KIR*-like sequences were also identified in the genome of Equids. Besides a previously identified non-functional KIR-Immunoglobulin-like transcript fusion gene (*KIR-ILTA*) and two putative pseudogenes, a *KIR3DL*-like sequence was analyzed. In contrast to previous observations made in the horse, the *KIR3DL* sequence, genomic organization and mRNA expression suggest that all Equids might produce a functional KIR receptor protein molecule with a single non-mutated immune tyrosine-based inhibition motif (ITIM) domain. No evidence for positive selection in the *KIR3DL* gene was found. Phylogenetic analysis including rhinoceros and tapir genomic DNA and deduced amino acid *KIR*-related sequences showed differences between families and even between species within the order *Perissodactyla*. The results suggest that the order *Perissodactyla* and its family *Equidae* with expanded *Ly49* genes and with a potentially functional *KIR* gene may represent an interesting model for evolutionary biology of NKR genes.

## Introduction

Natural killer (NK) cells have complex biological functions in both innate and adaptive immunity. They can recognize and subsequently eliminate microbe-infected and/or tumor cells, but they also have positive or negative influence on host T and B cell immunity. They express a repertoire of activating and inhibitory receptors (NKRs) and can produce various cytokines [1]. NK recognition in mammals can be mediated through highly variable killer cell immunoglobulin-like receptors (KIR) and/or killer cell lectin-like Ly49 receptors. Killer immunoglobulin-like receptors (KIRs) expressed on NK cells bind major histocompatibility complex (MHC) class I ligands. They may exist in two forms. KIR receptors with a long cytoplasmic tail deliver an inhibitory signal when bound to their ligands, while KIRs with a short cytoplasmic tail activate NK responses. The *Ly49* family encodes C-type lectin-like Ly49 molecules also interacting with classical MHC class I molecules, due probably to convergent evolution. This component of immune responses is very dynamic, subject to varying selection pressures [2]. NKR genes thus might be useful for understanding evolution and function of innate immunity [3]. NKR genes represent rapidly evolving genomic regions. No single conservative model of NKR genes was observed in mammals. Important interspecific differences in the usage of *KIR* and/or *Ly49* genes even within orders and families may be observed (reviewed in [4]). Single-copy low polymorphic NKR genes present in one mammalian species may expand into highly polymorphic multigene families in other species [5].

A highly polymorphic *Ly49* multigene family was identified in the mouse and *Ly49* genes are also present in multiple copies in the rat [6]. On the other hand, this gene exists as a single copy in baboons [7] and orangutans [8] and one non-functional copy was found in humans [9], where the *KIR* gene family expanded [4]. The expansion of the *KIR* family is not restricted to primates. It seems that cattle have a single *Ly49* [10] and multiple *KIR* genes [11], while a single *KIR* gene was found in pigs [12]. The domestic cat genome contains one *KIR3DL* gene with a frameshift mutation, while the dog genome lacks *KIR* sequences [13]. Intact open reading frames and a single immune tyrosine-based inhibition motif (ITIM) in the putative Ly49 proteins suggest that Ly49 in the domestic cat, dog, and pig could act as inhibitory NK receptors [14]. No species has yet been found to have both expanded and variable *Ly49* and *KIR* genes [2].

Domestic mammals represent suitable models for evolutionary biology in general [15]. Among them, the family *Equidae* consisting of a single genus, *Equus* [16] with different free-living and domesticated species exposed to a variety of pathogens in different habitats is a suitable model for analyzing diversity and evolution of immunity-related genes [17]. It is a rapidly evolving mammalian family, both at the karyotype [18] and molecular [19] level. Therefore, the *Equidae* might also be interesting models for studying evolution of NKR genes. Only limited information on *KIR*/*Ly49* genes in the domestic horse is available. In contrast to other mammals, several *Ly49*-like genes appear to exist in the horse, indicating amplification of this locus in a non-rodent lineage [20]. The horse has at least six *LY49* genes, five with an immunoreceptor tyrosine-based inhibition motif (ITIM) and one with arginine in the transmembrane region. None of the horse *KIR*-like cDNA clones analyzed in this study encoded molecules likely to be functional NK receptors. Four types of clones were KIR-Ig-like transcript (*KIR-ILT*) hybrids containing premature stop codons and/or frameshift mutations, and two putative allelic sequences predicting KIR3DL molecules had mutated ITIM. Radiation hybrid mapping and fluorescence in situ hybridization localized horse *LY49* and *KIR* genes to chromosomes 6q13 and 10p12, respectively [20]. No information on *KIR/Ly49* genes in other Equid species and on their evolution in this family is available, due also to the fact that assembled full genome sequences have not yet been published for these species.

The objective of this study was to study NKR genes and their evolution in the *Equidae* with special focus on *KIR* genes.

## Materials and Methods

### Ethical Statement

The work and sample collections were conducted in compliance with all national and international standards for animal welfare. All blood samples were originally collected for other purposes and shared as acknowledged at the end of the article. Samples from Camargue, and Murgese horses, as well as from all Perissodactyla kept in zoos were collected primarily for diagnostic purposes by licensed local and/or zoo veterinarians in compliance with all professional standards. Samples from Old Kladruber horses were collected by the local licensed veterinarian for the purposes of another project on genetic diversity and melanoma, approved by the Ethical Committee of the University of Veterinary and Pharmaceutical Sciences Brno, Czech Republic.

### Animals

One hundred twenty-six domestic horses of three breeds (40 Camargue, 42 Murgese, 44 Old Kladruber), and a panel of 20 zebras, donkeys and asses, including two individual of each species/sub-species was used for analysis. Zebras *Equus grevyi, Equus zebra hartmannae, Equus burchellii boehmi*, *Equus burchellii antiquorum, Equus burchellii chapmanni, Equus burchellii cunninghami,* and donkeys and asses *Equus asinus, Equus asinus somalicus, Equus kiang* and *Equus hemionus* kulan composed the panel.

For phylogenetic analyses, DNA from three rhinoceros (*Rhinoceros unicornis, Diceros bicornis, Ceratotherium simum*) and one tapir (*Tapirus terrestris*) species were used.

### Genomic DNA Extraction

Genomic DNA was extracted from EDTA-anticoagulated peripheral whole blood samples kept frozen at −20°C by using NucleoSpinBlood kit (Macherey-Nagel, Düren, Germany) according to the manufacturer’s protocol.

### Primers

All primers used in this study were designed by using the Primer-BLAST software [21] based on the horse genome assembly EquCab2.0 (GenBank GCA_000002305.1) [22].

### DNA Sequencing

Standard Sanger sequencing was provided on a commercial basis (Macrogen, Seoul, Korea; MWG Operon, Martinsried, Germany)**.** Next generation sequencing of all long-range PCR products and cDNAs was performed with the GS Junior Titanium Series 454 (Roche, Basilei, Switzerland) device. Rapid library preparation, emulsion PCR and sequencing were performed according to original manufacturer’s protocols. For each Equid species, two PCR products from individual animals were equimolarly combined and one library was prepared. Sequences were obtained by alignment of reads in GS Reference Mapper program v.2.7 (Roche –454 Life Sciences, Branford, USA) with the horse reference genomic sequences (all Equids) or, in case of rhinoceros, by assembly in GS De Novo Assembler program v.2.7 (Roche –454 Life Sciences, Branford, USA). Alignments were inspected and confirmed sequence variants were treated as polymorphisms and written to consensus sequences using IUPAC nucleotide ambiguity codes in BioEdit, version 7.0.9.0 [23].

### Expression of KIR3DL and KIR-ILTA

The mRNA expression of *KIR3DL* and *KIR-ILTA* was tested by reverse-transcription PCR (RT-PCR) in the horse and the donkey. Peripheral blood leukocytes were isolated according to Zizzadoro et al. [24] from 5 ml of fresh blood. 100 µl of the resulting leukocyte suspension in 0.9% NaCl were mixed with 900 µl of RN*Alater*® (Sigma-Aldrich, Saint Louis, MO, USA) and kept at 4°C until further processing. Total RNA was isolated from ∼12.5×10^6^ cells in 200 µl of phosphate buffered saline using HighPure RNA Isolation kit (Roche Applied Science, Mannheim, Germany) following the manufacturer’s protocol. RNA concentration was assessed by using Quant-iT™ RNA Assay Kit and Qubit® fluorometer (Invitrogen, Eugene, Oregon, USA).1 µg of purified total RNA was used for cDNA synthesis by using the QIAGEN® LongRange 2Step RT-PCR kit (Qiagen, Hilden, Germany).

Specific second-step PCR amplifications of *KIR3DL* (1448 nt) and *KIR-ILTA* (1218 nt) were performed in the 25 µl reaction volume with 2.5 µl of reverse transcription reaction (cDNA) as template using same primers as for genomic sequences (Table S1). The PCR protocol consisted of denaturation at 93°C for 3 min; 35 cycles of 15 sec at 93°C, 30 sec at 62°C and 1 min 30 sec at 68°C; and final cooling to 4°C. PCR products were used for 454 sequencing.

### Sequence Alignment

Sequences were aligned using CLUSTALW algorithm in BioEdit, version 7.0.9.0 [23] and manually adjusted to maximize alignments, if needed. Haplotypes for *KIR3DL* genomic sequences were reconstructed by using PHASE algorithm in DnaSP 5.10 program [25] and amino acid sequences from protein coding regions were deduced.

### Phylogenetic Analysis

Maximum Likelihood phylogenetic analysis was conducted with MEGA version 5 [26] for individual *KIR* genes and for exons of *Ly49* genes based on nucleotide sequences using the Hasegawa-Kishino-Yano model with 500 replicates. Amino acid sequences of KIR3DL and Ly49 homologues from selected mammals were aligned and the neighbor-joining phylogenetic tree was constructed based on the p-distance method with 1000 replicates in the MEGA v.5 program.

Analysis of positive selection was conducted in MEGA v.5 using HyPhy estimation of selection on each codon of Equid sequences encoding KIR3DL by testing the average rate of non-synonymous/synonymous substitution ratios in a group of 24 protein coding sequences derived from haplotypes inferred in DnaSP 5.10 program [25].

### Identification of Ly49 Genes in the Equidae

The presence of expanded *Ly49* genes in the *Equidae* was assessed by nested PCR. Long-range (LR) PCRs amplifying genomic DNA between the 5′-untranslated region and exon 2 (common for all genes), and between exon 4 and exon 6 (gene specific reverse primer in 3′UTR) were performed with the Verbatim High Fidelity DNA Polymerase (Thermo Fisher Scientific- ABgene UK, Epsom, United Kingdom) according to the manufacturer’s protocol. Gene specific nested/semi-nested PCRs amplifying individual exons were then carried out by the QIAGEN® HotStarTaq Master Mix (Qiagen, Hilden, Germany) in duplicate 12.5 µl reactions. For this purpose, primers specific for exon 1 encoding cytoplasmic tail and for exons 5 and 6, encoding the C-type lectin-like domain of the horse (*Equus caballus*) *LY49B, LY49D, LY49C, LY49E* and *LY49F* genes were used. Primer sequences, annealing temperatures and PCR product sizes are summarized in Table S1. *Ly49B, C, D, E,* and *F* genes were analyzed. Due to high sequence similarities, it was impossible to design locus-specific primers for *LY49A*. Therefore, the presence of this gene has not been investigated for the purposes of this study.

The long range PCR protocol consisted of initial denaturation 95°C for 3 min, followed by 25 cycles of 98°C for 20 s, annealing for 20 s and elongation at 72°C for 1 min 45 s (5′UTR-exon 2) or 3 min (exon 4-exon 6) and final extension at 72°C for 5 min. Protocols for nested PCR consisted of initial denaturation 95°C for 15 min, followed by 35 cycles of 94°C for 30 s, annealing for 20 s and elongation at 72°C for 30 s with final extension at 72°C for 10 min. All amplicons were checked by gel electrophoresis, purified by High Pure PCR Product kit (Roche Diagnostics, Mannheim, Germany) and commercially sequenced. The sequences retrieved were aligned and analyzed for sequence identity and polymorphisms in BioEdit, version 7.0.9.0 [23]. The Ly49 amino acid sequences were deduced from exon 5 and 6 sequences and a phylogenetic tree was constructed.

### “In silico” search for KIR-related Sequences in the Horse Genome

Previously reported horse *KIR-ILTA* mRNA sequence (GenBank accession number AB120396), *KIR3DL* mRNA (GenBank accession number AB120394), and model RNA sequences (3222264.m; 3274264.m) predicted by Gnomon were compared and used for the step-by-step BLAST search in the horse genome assembly EquCab2.0 (http://www.ncbi.nlm.nih.gov/blast/Blast.cgi?PAGE_TYPE=BlastSearch&PROG_DEF=blastn&BLAST_PROG_DEF=megaBlast&BLAST_SPEC=OGP__9796__11760).

The corresponding sequences retrieved from the horse genome along with their flanking sequences were compared by VISTA (www-gsd.lbl.gov/vista) [27]. Besides equine *KIR-ILTA* and *KIR3DL* sequences reported previously [20], two novel *KIR*-related sequences designated as *KIRP1* and *KIRP2* were identified in the equine genome.

The *KIR3DL* exon 9 nucleotide sequence and the deduced amino acid sequence of the cytoplasmic domain were used to search for further KIR-related molecules with ITIM domains, and for their genes.

### Molecular Analysis of Novel KIRP1 and KIRP2 Sequences

Two novel KIR-related sequences were annotated in the domestic horse full genome assembly based on sequence similarity to *KIR3DL*. The *KIRP1* and *2* sequences were amplified as 7261 bp and 6254 bp long fragments including putative exons by same LR-PCR protocol as *KIR-ILTA* (see below) with primers and annealing temperatures listed in Table S2. PCR products were used for massive parallel sequencing. Sequences were aligned and used for phylogenetic analysis.

### Molecular Identification of the KIR-ILTA Fusion Gene in Equid Species

Horse-specific primers (Table S2) located in exons 1 and 8 encompassing the entire *KIR-ILTA* gene (14341 bp) were used for a long-range PCR amplification and subsequent amplicon massive parallel sequencing of the panel of genomic DNAs from zebras, donkeys, asses and horses. Long-range PCRs were performed by using the Expand Long Range dNTPack (Roche Diagnostics, Mannheim, Germany) and DNA Engine (MJ Research, USA) thermocycler. PCR protocols consisted of initial denaturation at 92°C for 2 min, followed by 10 cycles of 92°C for 10 s, annealing for 15 s and elongation at 68°C for 60 s/kb and 25 cycles of 92°C for 10 s, annealing for 15 s and elongation at 68°C for 60 s/kb +20 s for each successive cycle with final elongation at 68°C for 7 min. Since the LR-PCR amplifications were not successful in all samples, the presence of the *KIR-ILTA* fusion was definitively determined by amplifying shorter *KIR-ILTA* sequences covering the exon 1– exon 6 (6653 bp) interval and containing the fusion of interest. The same exon 1 forward primer and an intron 6 reverse primer were used (Table S2). The sequences obtained from different groups of Equids were aligned and used for phylogenetic analyses. Expression of *KIR-ILTA* sequences in peripheral blood leukocytes of horses and donkeys was assessed by RT-PCR and resulting cDNA were sequenced.

### Characterization of Putative KIR3DL Orthologues in Equids

The genomic organization of the *KIR3DL* sequence in various species of the *Equidae* was determined based on massive parallel sequencing following its long-range PCR amplification from genomic DNA. The size of the PCR product was 9401 bp and it included the complete coding sequence (Table S2). The PCR protocol consisted of initial denaturation at 92°C for 2 min, followed by 10 cycles of 92°C for 10 s, annealing at 60°C for 15 s and elongation at 68°C for 9 min 20 s and 25 cycles of 92°C for 10 s, annealing for 15 s at 60°C and elongation at 68°C for 9 min 20 s/+20 s for each successive cycle with final elongation at 68°C for 7 min. PCR products were checked, quantified and kept frozen at −20°C until 454 sequencing. The sequences retrieved were aligned and analyzed. A phylogenetic tree was constructed and analysis of positive selection was performed. Expression of *KIR3DL* sequences in peripheral blood leukocytes of horses and donkeys was assessed by RT-PCR and resulting cDNA were sequenced.

Two non-synonymous single nucleotide polymorphisms identified *in silico* based on GenBank sequences in exon 3 (1276G→A) and exon 5 (4451G→A) of the horse *KIR3DL* gene, and a putative insertion in exon 9 (9208insC) of sequence reported previously by Takahashi et al. [20] (GenBank: AB120394) were analyzed in three unrelated horse breeds: Camargue (40 horses), Murgese (40 horses) and Old Kladruber (44 horses). The QIAGEN® HotStarTaq Master Mix (Qiagen, Hilden, Germany) was used to amplify selected parts of the gene containing the polymorphisms sought in a nested PCR (primers, regions amplified, PCR product sizes and annealing temperatures are in Table S2). The *KIR3DL* 9.4-kb amplicon was used as template in three separate nested PCR. Protocols for nested PCR consisted of initial denaturation 95°C for 15 min, followed by 35 cycles of 94°C for 30 s, annealing at 60°C for 20 s and elongation at 72°C for 30 s (exon 3–380 bp; exon 5–91 bp ) or 50 sec (exon8-exon9–855 bp) with final extension at 72°C for 10 min.

For the purposes of population analysis, exon 3 SNP (164 G/A), exon 5 SNP (48 G/A), and exon8-exon9 indel (641 in/del C) were genotyped by PCR-RFLP with appropriate restriction endonucleases (BsrI, NcoI and Hpy188I, New England Biolabs, USA). Fragments were resolved and read by using the chip capillary electrophoresis (MultiNA, Shimadzu, Kyoto, Japan).

### Search for the KIR-ILTA and KIR3DL Genes in other Perissodactyla

The presence of *KIR-ILTA-*related sequences in genomes of rhinoceros and tapir species was tested by amplifying the fusion-containing region (1505 bp) with primers located in intron 5 and intron 6 (Table S2) and with the QIAGEN® HotStarTaq Master Mix (Qiagen, Hilden, Germany) in PCRs composed of initial denaturation 95°C for 15 min, followed by 35 cycles of 94°C for 30 s, annealing at 60°C for 20 s and elongation at 72°C for 1 min 30 s with final extension at 72°C for 10 min.

*KIR3DL* sequences were amplified in two overlapping long-range PCRs. First fragment covering exon3-exon5 (3329 bp) was amplified using primers kir3dl ex3 fw and kir3dl ex5 rev (Table S2) in Expand LongRange dNTPack system (Roche Diagnostics, Mannheim, Germany) with initial denaturation at 92°C for 2 min, followed by 10 cycles of 92°C for 10 s, annealing at 60°C–55°C (touchdown 0,5°C per cycle) for 15 s and elongation at 68°C for 5 min and 25 cycles of 92°C for 10 s, annealing for 15 s at 55°C and elongation at 68°C for 5 min/+20 s for each successive cycle with final elongation at 68°C for 7 min. Second fragment covering exon5–exon9 (5020 bp) was amplified by same protocol with both annealing temperatures at 50°C and primers in exon 5 (kir3dl ex5 fw) and 3′UTR (kir3dl rev). PCR products were then used for ***454 sequencing.***

A phylogenetic tree including selected KIR3DL homologous sequences identified in other mammals was constructed.

## Results

### Identification of LY49 Genes in the Equidae

**Figure 1:**
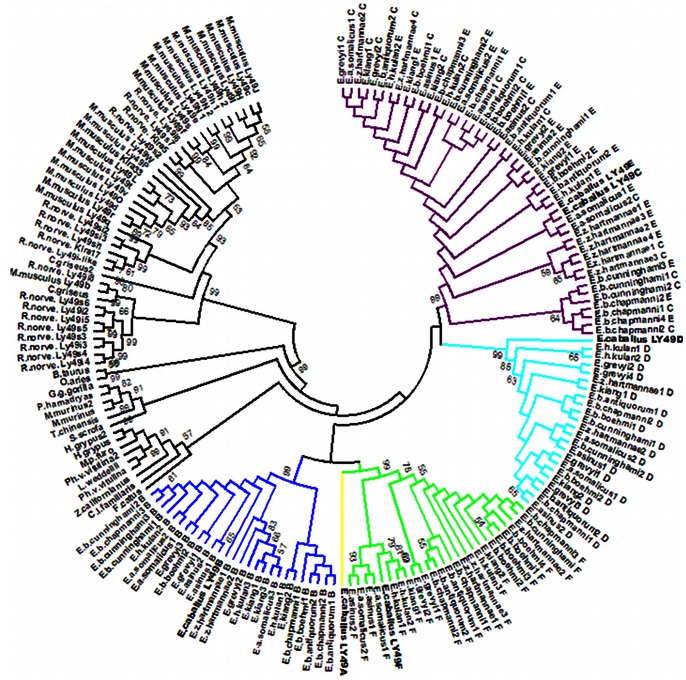
Phylogenetic tree of mammalian Ly49 C-type lectin-like domain sequences. The evolutionary history was inferred using the neighbor-joining method [39]. The bootstrap consensus tree inferred from 1000 replicates [40] is taken to represent the evolutionary history of the taxa analyzed: *Mus musculus* (NP_032489.1, NP_032490.1, NP_444384.1, NP_034781.2, NP_034778.2, NP_032485.2, NP_444381.1, NP_001095090.1, NP_032487.2, NP_034780.1, NP_001239078.1, NP_038821.2, NP_444382.1, NP_444380.1, NP_034776.1, NP_077790.1, NP_444383.1, NP_001034207.1, NP_001239506.1, NP_573466.3, NP_038822.3, NP_032488.4); *Rattus norvegicus* (NP_001009718.1, NP_775413.1, NP_942041.1, NP_001009494.1, NP_001009919.1, NP_001009497.1, NP_001009498.1, NP_001104780.1, NP_001165559.1, NP_001009495.1, NP_001009499.1, NP_714948.1, NP_001012767.1, NP_001009487.1, NP_690061.1, NP_001009501.1, NP_001009486.1, NP_001009488.1); *Cricetulus griseus* (XP_003510604.1, XP_003509313.1); *Bos taurus* (NP_776801.1); *Ovis aries* (XP_004006913.1); *Gorilla gorilla gorilla* (XP_004052762.1); *Papio hamadryas* (AAK26161.1); *Microcebus murinus* (ACO83129.1, ACO83128.1); *Tupaia chinensis* (ELV12449.1); *Sus scrofa* (AAP13541.1); *Halichoerus grypus* (ACN78613.1, ACN78614.1); *Mustela putorius furo* (AES00881.1); *Phoca vitulina vitulina* (ACN78615.1, ACN78616.1); *Leptonychotes weddellii* (ACN78617.1); *Zalophus californianus* (ACN78618.1); *Canis lupus familiaris* (AAP13540.1); *Felis catus* (AAP13539.1); *Equus caballus* (NP_001075297.1, NP_001075298.1, NP_001075299.1, NP_001075392.1, NP_001075393.1, NP_001075998.1); *Equus grevyi*; *Equus zebra hartmannae*; *Equus burchellii boehmi*; *Equus burchellii antiquorum*; *Equus burchellii chapmanni*; *Equus burchellii cunninghami*; *Equus asinus*; *Equus asinus somalicus*; *Equus kiang* and *Equus hemionus kulan*. Bootstrap confidences over 50% are given as numbers. The evolutionary distances were computed using the p-distance method [41]. The analysis involved 184 amino acid sequences. All ambiguous positions were removed for each sequence pair. A total of 135 positions were included into the final dataset. Evolutionary analyses were conducted in MEGA5 [26].

Expansion of *LY49* genes was observed in the genomes of all Equids analyzed. Based on exon 1, exon 5 and exon 6 sequences amplified with horse primers, all five *Ly49* genes (*Ly49B, C, D, E,* and *F*) investigated were identified in all species. High sequence similarities among Equid species as well as inter- and intra-species polymorphisms were observed (Table 1). Exon 5 and 6 sequences encoding the C-type lectin-like domain contained more polymorphic nucleotide positions than those of the cytoplasmic domain. Phylogenetic relationships of the Equid C-type lectin-like domain amino acid sequences with known mammalian Ly49 receptors are in Figure 1. The tree shows clear separation of the *Equidae* from other ungulates and carnivores with single *Ly49* genes as well as from rodents with expanded *Ly49* family genes. Within the family *Equidae*, extensive sharing of the C-type lectin-like domain sequences can be observed between species and even between Ly49C (activating) and Ly49E (inhibitory) receptors.

**Table 1:** Number of interspecies/intra-species polymorphisms of *LY49* genes in the *Equidae.*

Gene	exon1^a^	exon5^b^	exon6^b^
*LY49C*	0/0	1/1	0/2
*LY49E*	4/1	0/1	1/1
*LY49B*	4/0	1/10	8/3
*LY49D*	1/0	2/3	9/1
*LY49F*	?^c^	5/5	6/4
***total***	9/1	9/20	14/11

a Coding for the cytoplasmic tail.

b Coding for the C-type lectin-like domain interacting with major histocompatibility complex type I molecules.

c could not be determined due to high sequence similarities with *LY49A* sequences.

### “In silico” search for KIR-related Sequences in the Horse Genome

The presence of previously reported *KIR-ILTA* and *KIR3DL* sequences was confirmed in the full genome sequence assembly EquCab2.0. In addition, two *KIR-*related sequences were identified based on their 45% and 40% sequence identities to the equine genomic *KIR3DL* sequence and high similarity in exon-intron organization as revealed by VISTA alignment (Figure S1). The *KIR-ILTA* genomic sequence differed from the sequence reported by Takahashi et al. [20] (AB120396) by eight single nucleotide polymorphisms and three indels. The *KIR3DL* differences included insertion in exon 9 (9208insC) in the sequence reported previously by the same authors (AB120394) compared to genome assembly and two single nucleotide polymorphisms.

The BLAST search of horse reference proteins revealed members of the leukocyte immunoglobulin-like receptor (*LILR*) family with identical (XP_001488598.3, XP_001488973.3, XP_001917760.2) or with highly similar ITIM motifs (XP_001489220.2, XP_001491661.3, XP_001494763.2) in the cytoplasmic domain. All these *LILR* DNA genomic sequences are located in the vicinity of other *KIR-*related genes on the horse chromosome 10 (ECA10).

### Novel KIRP1 and KIRP2 Sequences

**Figure 2:**
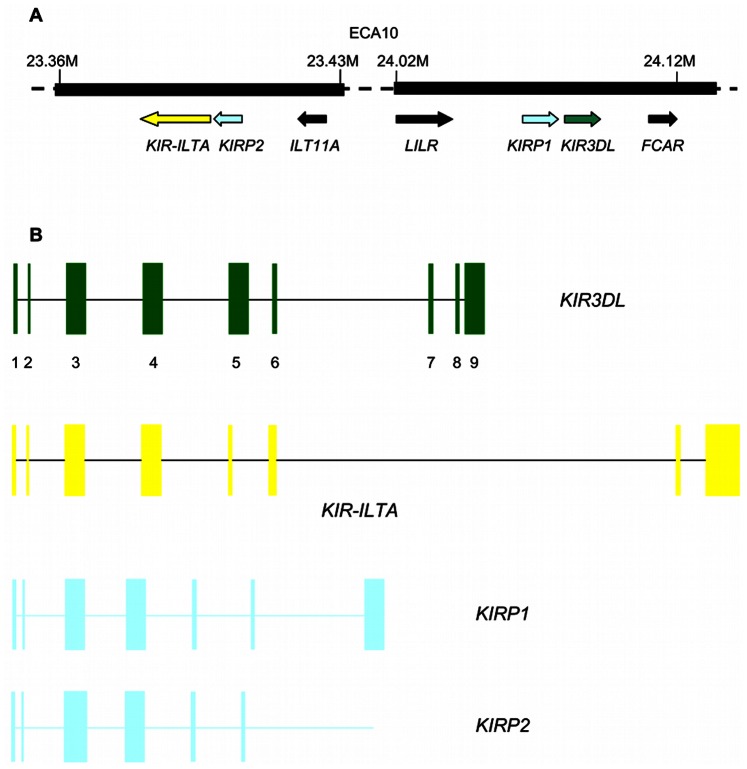
Genomic organization of *KIR*-related sequences on the horse chromosome 10. **A** Mirror-faced, head-to-tail oriented *KIR*-related sequences (based on the horse genome assembly EquCab2.0) **B** Signal peptid (1,2), D0 (3), D1 (4), D2 (5), stem (6), stem/transmembrane (7), and cytoplasmic tail (8,9) domains of the KIR3DL deduced protein represented as coding exons.

The two novel sequences contain premature stop codons and/or frameshift mutations and can be considered as pseudogenes. Therefore, they were designated as *KIRP* (*KIR* pseudogenes). Their position relative to *KIR3DL* and *KIR-ILTA* genes and their putative exons are shown in Figure 2. They were found in all Equids (GenBank accession numbers KC315971-KC315981 for *KIRP1* and KC315982–KC315992 for *KIRP2*). Similarities to the horse sequence varied among species from 97.36% to 97.96% (Table 2); they ranged from 69.74% to 70.43% between the two genes within one species. There were 264 and 242 variable sites in the *KIRP1* and *KIRP2* sequences among the Equids analyzed, respectively. The phylogenetic trees for these sequences in the family *Equidae* are in Figure S2.

**Table 2:** Sequence identity (%) of *KIR*-related sequences from different Equids with the reference horse sequence.

	*Equus* *grevyi*	*Equus zebra* *hartmannae*	*Equus burchellii* *antiquorum*	*Equus burchellii* *boehmi*	*Equus burchellii* *chapmani*	*Equus burchellii* *cunninghami*	*Equus asinus* *somalicus*	*Equus* *asinus*	*Equus* *kiang*	*Equus hemionus* *kulan*
***KIR3DL***	99.25	99.22	99.16	99.18	99.22	99.09	99.20	99.12	99.00	99.08
***KIR-ILTA***	98.08	98.01	98.05	97.96	97.93	97.93	97.72	97.69	98.13	98.07
***KIRP1***	97.92	97.93	97.81	97.69	97.65	97.87	97.92	97.96	97.36	97.79
***KIRP2***	97.63	97.68	97.67	97.59	97.63	97.73	97.84	97.54	97.83	97.71

### Molecular Identification of the KIR-ILTA Fusion Gene in Equid Species

LR-PCR amplified successfully the complete genomic sequence coding KIR-Ig-like fusion transcript (*KIR-ILTA*) originally reported by Takahashi et al. [20] from the domestic donkey (*Equus asinus,* GenBank: KC412058) and in Burchell’s zebra (*Equus burchellii antiquorum,* GenBank: KC412059). Their sequence similarities to the horse genome sequence were 98.08% and 98.46%, respectively. In other Equids from the panel, amplifications failed.

When analyzed by shorter LR-PCR, the presence of the fusion containing *KIR-ILTA* exon 1–exon 6 sequence was confirmed (GenBank accession numbers KC315960–KC315970). Sequence similarities with the horse ranged from 97.69% to 98.13% (Table 2), and 195 nucleotide positions variable between species were observed. Transcription of *KIR-ILTA* was confirmed in horse (KC412062) and donkey (KC412063) peripheral blood leukocytes. The cDNA sequence identity between the two species was 98.53%. The phylogenetic tree constructed for the family *Equidae* is in Figure S3.

### Characterization of Putative KIR3DL Orthologues in Equids

**Figure 3:**
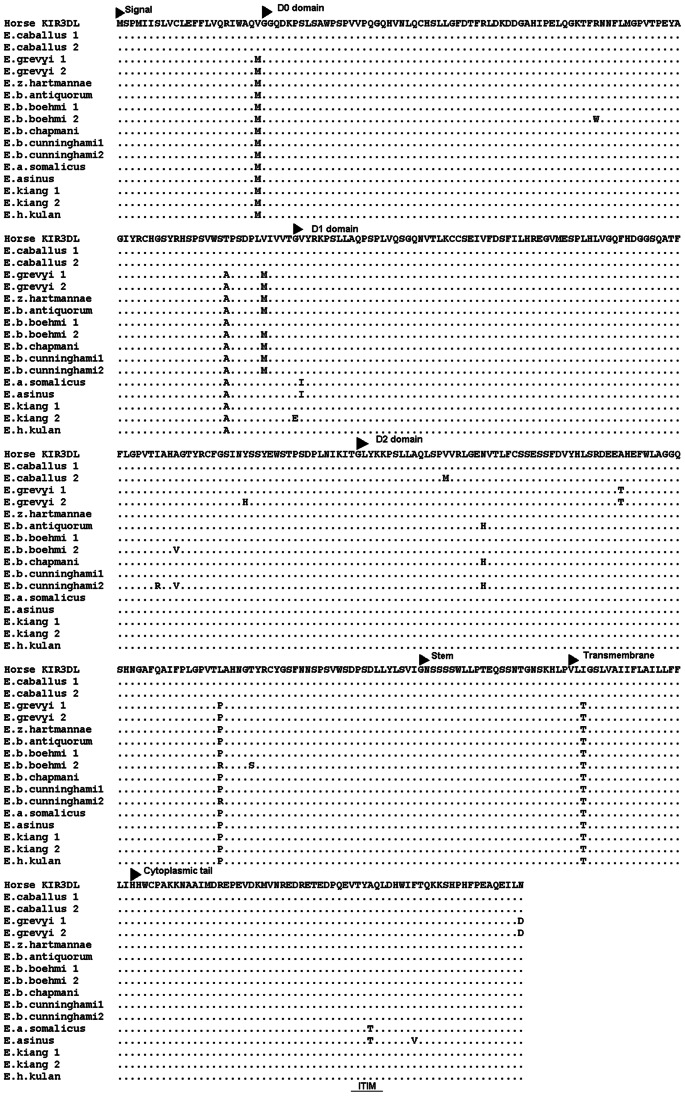
Deduced amino acid sequences of KIR3DL molecules in the *Equidae.* All sequences were deduced from genomic *KIR3DL* sequences (GenBank accession numbers KC315949–KC315959), except the horse sequence deduced from the cDNA sequence reported here (GenBank: KC412060).

Genomic sequences similar to the horse *KIR3DL*-like transcripts (AB120394, AB120395) reported by Takahashi et al. [20] were identified in all Equids (GenBank accession numbers KC315949–KC315959). However, no mutation of the ITIM domain was observed in genomic sequences from 3 horses, 9 asses and 12 zebras. Furthermore, insertion within the ITIM domain was not found by PCR-RFLP analysis of 124 horses of three breeds (see below). One complete ITIM motif was found in orthologous sequences in the entire family. Based on its genomic structure and 99% identity with the horse sequence (Table 2), the sequence was annotated as a *KIR3DL* gene with structural features of a functional gene. Interspecific differences and within-species SNPs were observed at 197 positions. Thirty-two of them were located in the coding region and 19 were non-synonymous (Figure 3). Analyses of the average rate of non-synonymous/synonymous substitutions ratio did not identify any amino acid position in the KIR3DL putative protein that could be under positive selection. The sequence was expressed at the mRNA level in peripheral blood leukocytes of domestic horse (KC412060) and donkey (KC412061). Their identity was confirmed by next generation sequencing; the two cDNA sequences were 99.03% identical. The phylogenetic tree is similar to that reported in Equids for other coding genes (Figure S3).

Allele frequencies of two single nucleotide polymorphisms are summarized in Table 3. Notable interbreed differences were observed, suggesting existence of various haplotypes of *KIR3DL* in real horse populations.

**Table 3:** Minimum allele frequencies of *KIR3DL* single nucleotide polymorphisms in three horse breeds.

Breed	1276 G→A (61 Asp→Asn)	4451 G→A (233 Val→Met)
	exon 3	exon 5
Camargue (N = 40)	A 0.184	A 0.366
Murgese (N = 40)	A 0.075	A 0.288
Old Kladruber (N = 44)	A 0.148	A 0.000

### KIR-ILTA and KIR3DL Genes in other Perissodactyla

PCR of the fusion containing region (expected size 1505 bp) of the *KIR-ILTA* sequence with equine primers produced no amplicons in rhinoceros or tapir.

Exon 3– exon 9 sequences of *KIR3DL* were retrieved from two rhinoceros species – Indian (*Rhinoceros unicornis*) and white rhinoceros (*Ceratotherium simum*). The Indian rhinoceros *KIR3DL* (GenBank KC412055) contains two ITIM domain sequences located within the cytoplasmic tail of the putative protein. One potentially functional and one mutated (Leu to Phe substitution) ITIM domain was found in the white rhinoceros *KIR3DL* sequence (GenBank KC412056). Sequence identity between these two species was 91.22%. Sequence identity with the horse *KIR3DL* gene was 76.16% and 79.62% for Indian rhinoceros and for the white rhinoceros, respectively. A *KIR3DL*-like sequence was also amplified from the white rhinoceros genomic DNA (GenBank KC412057) by PCR for exons 3 to 5. The PCR protocols used could not amplify *KIR* genes in the black rhinoceros and/or in tapir.

**Figure 4:**
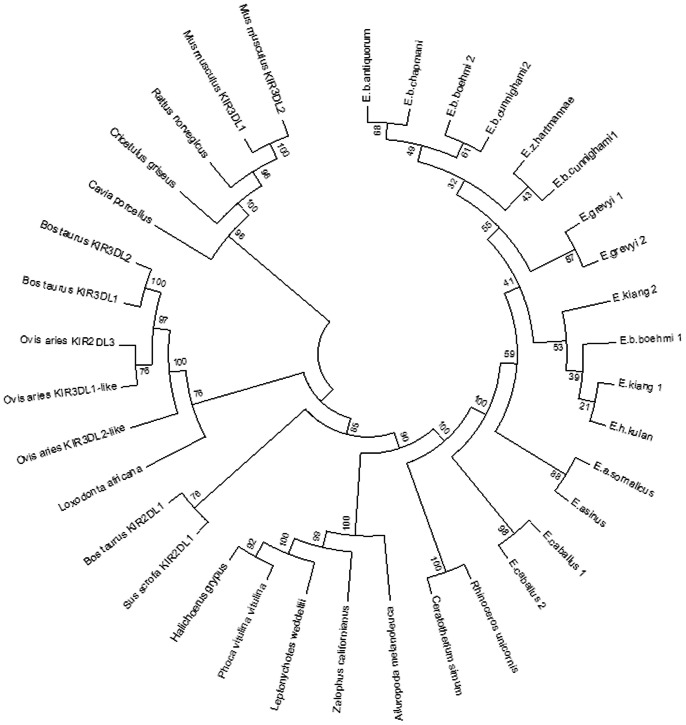
Phylogenetic tree of mammalian KIR3DL homologous sequences. Sixteen equid KIR3DL amino acid sequences were deduced from inferred haplotypes of genomic sequences (Accession numbers in Table S3). The tree was constructed by using the neighbor-joining method [39]. The evolutionary distances were computed using the p-distance method [41]. The analysis involved 36 sequences. All ambiguous positions were removed for each sequence pair. There were a total of 619 positions in the final dataset. Evolutionary analyses were conducted in MEGA5 [26].

Phylogenetic tree constructed for selected mammalian KIR proteins is in Figure 4. It shows clear separation of *Equidae* from carnivore sequences and close relation to *3DL*-lineage of ungulates, represented by cattle and pig KIR2DL1 amino acid sequences. It is evident that two rhinoceros sequences also belong to the *3DL*-lineage and are closely related to sequences obtained from the family *Equidae*.

## Discussion

Two types of mammalian NK receptors were reported in mammals. This study confirmed and extended previous findings by Takahashi et al. [20] showing that expansion of *LY49* genes occurred not only in the domestic horse but in the entire family *Equidae*. The structure of the *LY49* genomic region and its high sequence similarity to the rodent models suggested that Ly49 receptors might be fully functional in this mammalian group. Phylogenetic trees based on individual exons of *LY49* genes differ from the trees based on neutral markers. The sequences retrieved were highly similar each to the other and even shared by various species (Futas et al. in preparation). These data suggest possible effects of positive selection. Due to their complex genetic structure, diversity in individual Equid species, evolution, selection, and gene expression, the *LY49* genes will be characterized separately (Futas et al., in preparation). It is evident from the results presented here that multiple polymorphic *LY49* genes can be detected in the genomes of all Equids. This finding is important in the context of our analysis of the *KIR* genetic region.

The results showed that at least one functional and variable *KIR* gene may exist in the *Equidae.* Its genomic structure and the structure of the putative protein predicted from the cDNA sequence obtained are in agreement with this assumption. With one complete non-mutated ITIM motif, the gene is a representative of *KIR* genes with long cytoplasmic tails delivering inhibitory signals. The discrepancy between our results and those reported by Takahashi et al. [20] is due to a one-base difference in the nucleotide sequence – insertion of cytidine altering the ITIM motif. Since we have found the non-mutated sequences in the genomes of all horses, including the full-genome assembly, and of all Equids analyzed, it seems that it is the functional sequence, which is common in this family. The same sequences were also identified at the mRNA/cDNA level. As it was impossible to collect fresh blood samples from zebras and/or captive asses for the RT-PCR analysis, we could provide this evidence only for domestic horses and donkeys. In all mammalian species except mouse, the *3DL* - lineage *KIR* genes are flanked on one side by *FCAR*, a gene encoding the IgA receptor of myeloid cells (FcαRI), and on the other by the leukocyte immunoglobulin-like receptor (LILR) gene family. Since it is the same situation like in the horse genome (EquCab2.0 [22]), we believe that the gene detected is a true orthologue of functional *KIR* genes of other species. Its functional importance in Equids merits to be further investigated, although no signature of positive selection was found in the sequence analyzed.

In primates, the *KIR3DL1* gene is a functionally important gene, highly variable and interacting with MHC class I molecules [28]. Conserved genomic location of the horse *KIR3DL*, its high homology with the sequences retrieved from other Equids and other mammalian species, presence of functionally important motifs, and properties of the deduced protein provide another support for the hypothesis of a functional *KIR* gene in this family.

The KIRs are known to co-evolve with their MHC class I ligands [2]. The assembled genomic sequence of the horse MHC is similar to its human counterpart with an exception of a large segmental duplication found also in other Perissodactyla [29]. Seven out of 15 MHC class I loci identified in horses were expressed as mRNA [30]. Extensive polymorphism and variation in the number of expressed MHC class I loci per haplotype were observed [31]. Little information is available on functional interactions between KIR and MHC class I molecules in horses. MHC class I molecules are expressed at high levels on the equine chorionic girdle trophoblast cells [32]. These cells were susceptible to lymphokine-activated killer cell activity *in vitro* [33] and NK cells were identified in equine endometrial cups [34]. However, the role of KIR-MHC class I interactions in equine pregnancy and in other processes still remains to be studied.

Horses and other Equids thus seem to be species with expanded functional and variable *Ly49*, and at least one expressed *KIR* gene. It has been suggested that the ancestral state of these two gene families in placental mammals represent single functional *Ly49* and *KIR3DL* genes observed in pinnipeds [13]. The *Equidae* thus could be a group where both ancestral genes remained functional, with expansion of the *Ly49* family. This is in agreement with conserved genomic organization of both *KIR* and *Ly49* genomic regions. Genes and corresponding proteins belonging to the immunoglobulin gene superfamily encoded within the human KIR region on HSA19q are similar to those identified on the horse chromosome ECA10. Especially leukocyte immunoglobulin-like receptor (*LILR*) genes involved in regulating important immune functions show even more conservation than KIR receptors [35]. Our results show that similarly to rodents, primates, seals, chickens and recently cattle [36], *LILR* homologous sequences can also be found in the equine genome, showing extensive evolutionary conservation of the *KIR*-related region in these mammals.

However, the *KIR-ILTA* fusion gene had been found so far only in the domestic horse. Such a gene is not present in other ungulates; we were unable to find it in other Perissodactyla with horse primers. This however might be due to their lower (so far unknown) sequence similarity. We showed that the fusion must have occurred before speciation within the family *Equidae* in a common ancestor, and that it spread with only minor changes throughout the entire family. Evolution occurring in the *KIR* region of Equids is also documented by the presence of two pseudogenes, again in the entire family. Their origin is not clear. If really the ancestral state is represented by a single *KIR* gene, these pseudogenes as well as the *KIR-ILTA* must have resulted from gene duplications without functional effects. On the other hand, *in silico* translation from *KIRP1* genomic sequence shows maximum similarity to *3DX*-lineage of human, primates and cattle *KIR* genes (data not shown) and it thus could represent remnants of this lineage in Equids.

The *KIR*-related sequences retrieved from the *Equidae* seem to be rather conservative. All analyzed members of the family have a potentially functional *KIR3DL* gene, two *KIR* pseudogenes and a *KIR-ILTA* hybrid gene. Observations on a rapid karyotype evolution [18] supported by data on rapid evolution of insertion sequences [19] suggested that genomes of the *Equidae* are rapidly evolving. Within immunity-related genes, NKR genes belong to a very variable and little conservative group [2]. Therefore, high variability in NKR genes within this group could be expected. Nevertheless, the *KIR*-related sequences are rather conservative in terms of their presence in different species as well as in terms of sequence similarities of individual genes. It thus seems that this part of the genome of the family *Equidae* is more conservative than in other mammalian families studied so far, especially primates. These assumptions are further supported by our findings of rather high variation in *KIR* genes in rhinoceroses. Different species of rhinoceroses have different *KIR3DL* genes, with one or two potentially functional ITIM domains.

The phylogenetic trees similar to trees based on other expressed genes [37] or SNP markers [38] in Equids do not suggest effects of selection on evolution of this particular part of the *KIR* genomic region. The tree constructed with deduced protein sequences showed relationships to other ungulates. However, comparisons with cattle and pig *KIR* genomic region with no hybrid fusion gene and no annotated pseudogenes suggest that despite evolutionary relationships with Artiodactyls, specific evolution of *KIR* genes occurred in the family *Equidae*. Comparative *in silico* analysis of the equine *Ly49* genetic region on the chromosome ECA6 showed its high homology with the Natural Killer Complex (NKC) region on the human chromosome HSA12. Both regions contain the C-type lectin domain gene family along with genes encoding e.g. the tapasin B and CD4 proteins. Similarly to other mammals and different from birds, the NKC lineage was not maintained in the MHC. In contrast to the *KIR3DL*, the phylogenetic trees constructed for *Ly49* genes suggest possible effects of positive selection, which is in line with their potential functional importance. The *Equidae* thus represent an interesting model for comparative genomic and evolutionary biology of mammals.

## Supporting Information

Figure S1 **VISTA plot of horse** ***KIR3DL, KIR-ILTA*** **,** ***KIRP1*** **and** ***KIRP2*** **.** Genomic sequences flanked by 2 kb from both sides were compared using MLAGAN algorithm. Conserved regions with more than 70% sequence similarity over a 100 base pair window are colored: non-coding sequences apricot, exons purple, untranslated regions light blue. *KIR3DL* region was analyzed for the presence of long interspersed repeats (shown in red), short interspersed repeats (shown in green) and long terminal repeats (pink) or different repeats (olive) known from cow genome.
(TIF)

Figure S2 **Phylogenetic trees for equid genomic sequences of** ***KIRP1*** **and** ***KIRP2*** **.** The bootstrap confidence level of nodes is given in percentage as numbers (500 replicates). Accession numbers for *KIRP1* sequences are KC315971–KC315981 and for *KIRP2* KC315982–KC315992. Horse reference denotes corresponding genomic sequences as retrieved from horse genome assembly EquCab2.0.
(PDF)

Figure S3 **Phylogenetic trees for equid genomic** ***KIR3DL*** **(Accession numbers KC315949–KC315959) and** ***KIR-ILTA*** **(KC315960–KC315970) sequences.** The bootstrap confidence level of nodes is given in percentage (500 replicates). Horse reference refers to genomic sequences from the horse genome assembly EquCab2.0.
(PDF)

Table S1 **Primer sequences and annealing temperatures used for analysis of** ***LY49*** **genes.**
(PDF)

Table S2 **Primer sequences and annealing temperatures used for analysis of** ***KIR*** **-related sequences.**
(PDF)

Table S3 **List of accession numbers of mammalian KIR3DL sequences used for the phylogenetic tree construction.**
(PDF)
