# Supplementary figures and images for: Natural Killer Cell Receptor Genes in the Family Equidae: Not only Ly49
Source: PLoS One. 2013 May 28;8(5):e64736. doi: 10.1371/journal.pone.0064736 (PMC3665701; doi:10.1371/journal.pone.0064736)

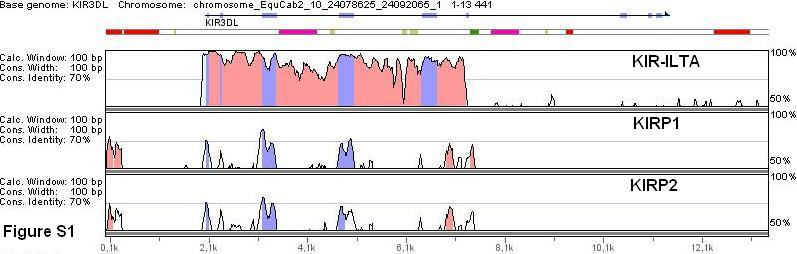

Supplement: Figure S1 — VISTA plot of horse KIR3DL, KIR-ILTA , KIRP1 and KIRP2 . Genomic sequences flanked by 2 kb from both sides were compared using MLAGAN algorithm. Conserved regions with more than 70% sequence similarity over a 100 base pair window are colored: non-coding sequences apricot, exons purple, untranslated regions light blue. KIR3DL region was analyzed for the presence of long interspersed repeats (shown in red), short interspersed repeats (shown in green) and long terminal repeats (pink) or different repeats (olive) known from cow genome. (TIF) [file pone.0064736.s001.tif]

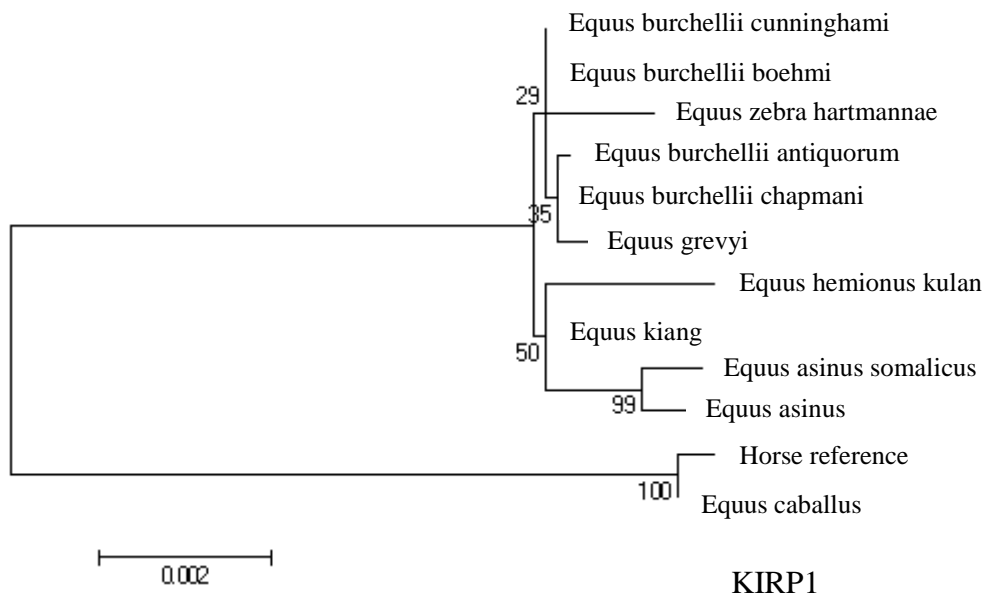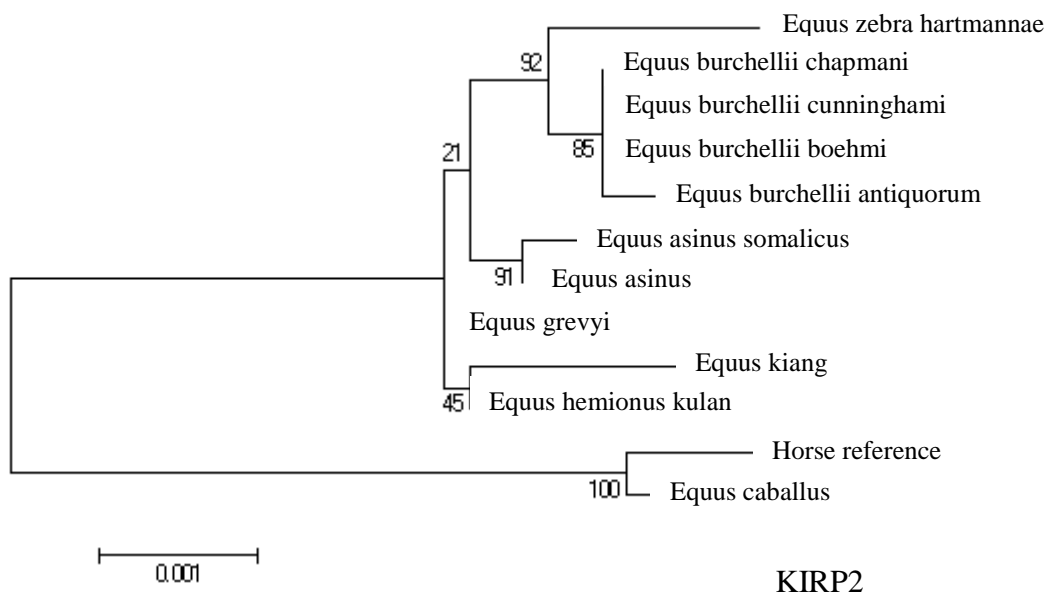

**Figure S2**

Supplement: Figure S2 — Phylogenetic trees for equid genomic sequences of KIRP1 and KIRP2 . The bootstrap confidence level of nodes is given in percentage as numbers (500 replicates). Accession numbers for KIRP1 sequences are KC315971–KC315981 and for KIRP2 KC315982–KC315992. Horse reference denotes corresponding genomic sequences as retrieved from horse genome assembly EquCab2.0. (PDF) [file pone.0064736.s002.pdf]

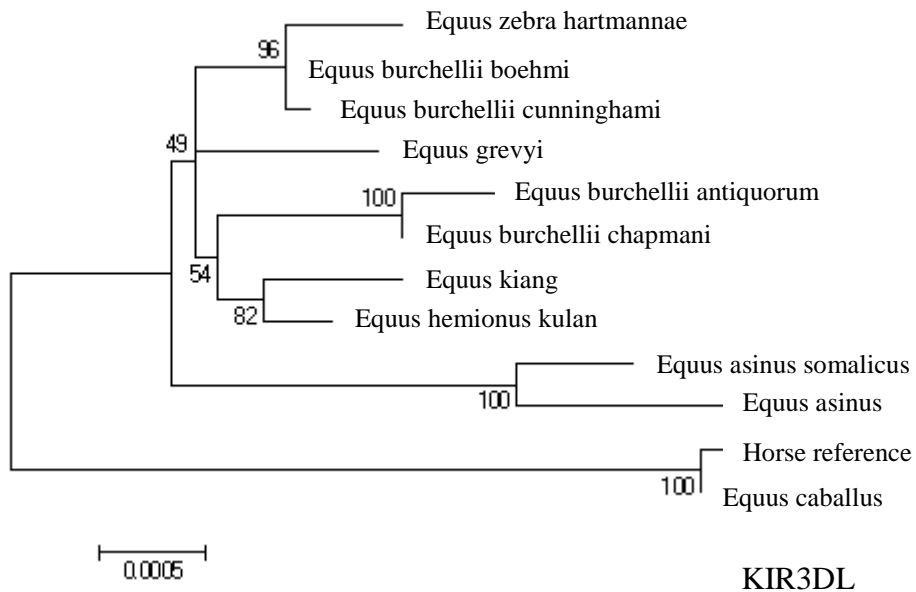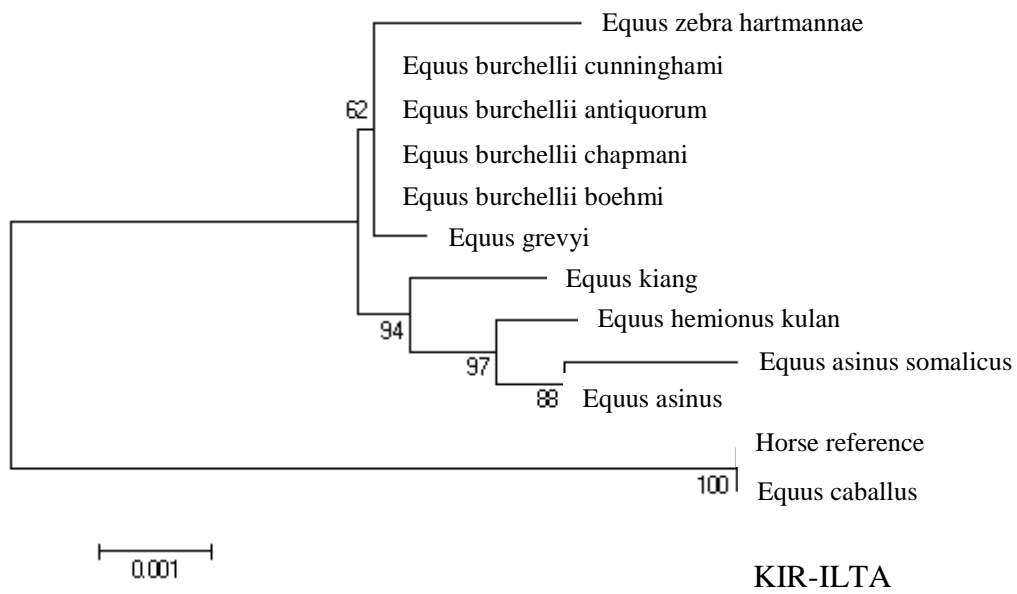

**Figure S3**

Supplement: Figure S3 — Phylogenetic trees for equid genomic KIR3DL (Accession numbers KC315949–KC315959) and KIR-ILTA (KC315960–KC315970) sequences. The bootstrap confidence level of nodes is given in percentage (500 replicates). Horse reference refers to genomic sequences from the horse genome assembly EquCab2.0. (PDF) [file pone.0064736.s003.pdf]
