# Supplementary material for: Natural Killer Cell Receptor Genes in the Family Equidae: Not only Ly49
Source: PLoS One. 2013 May 28;8(5):e64736. doi: 10.1371/journal.pone.0064736 (PMC3665701; doi:10.1371/journal.pone.0064736)
Supplement: Table S1 — Primer sequences and annealing temperatures used for analysis of LY49 genes. (PDF) [file pone.0064736.s004.pdf]

**Table S1**

| Gene/fragment | 5´-3´ sequences of primers forward/reverse | length of amplicon | annealing temperature |
|---|---|---|---|
| *LY49* universal 5´UTR-exon2 | ACCTTGTTGTTGGGTCAGCT/ CAGAGRATCCCAAGAGTCACTAC | app.2950-3200bp | 65°C |
| *LY49B* exon 1 | AGACATGAGCAATCARGAAGT/ TGTATATTCTAGTTACCGTCTAGTTATAAGT | 553 bp | 61°C |
| *LY49C* exon 1 | AGACATGAGCAATCARGAAGT/ ATTCATTAATTTGGAGAAATGGAGA | 305 bp | 61°C |
| *LY49D* exon 1 | AGACATGAGCAATCARGAAGT/ TTTGATTTCTCATTCTTTAAGACTTATG | 718 bp | 61°C |
| *LY49E* exon 1 | AGACATGAGCAATCARGAAGT/ GAGCAGGCCAAACTGTAATC | 422 bp | 61°C |
| *LY49F* exon 1 | AGACATGAGCAATCARGAAGT/ AACCTAGACGGCCAAAGCAG | 427 bp | 61°C |
| *LY49* exon4-exon6 | TGGTCCTGTTGTGGAGTAAACTG/ according to gene; see exon 6 reverse primers | various max.5,6kb | 60°C |
| *LY49B* exon 5 | GGAAGGTTTTCTAGGAAGGAAG/ TCTTACCAGTGACGTCTGCTT | 377 bp | 58°C |
| *LY49C* exon 5 | AAGAGAAGGTTTTTTAGGAAGGAGT/ CTTATTCTTGCCAACGAACTCTACT | 387 bp | 61°C |
| *LY49D* exon 5 | AGAGAAGGTTTTTTAGGAAGGAGG/ CGTATTCTTACCAATGACCTCTACCT | 389 bp | 58°C |
| *LY49E* exon 5 | AAGAGAAGGTTTTTTAGGAAGGAGT/ CTTATTCTTGCCAATGAACTCTACC | 388 bp | 61°C |
| *LY49F* exon 5 | GAGAGAAGGTTTTCTAGGAAGGAGA/ CTTATTCTTACCAATGACCTCTGCTTC | 388 bp | 58°C |
| *LY49B* exon 6 | GGTGTGATCAGGAGGCTGTTA/ AGGGCCATTAAAGAGATGGGA | 577 bp | 61°C |
| *LY49C* exon 6 | GGGTGTGATCAGAAGGCTGT/ CAGGGCCATTAAAGAGAAGAGC | 583 bp | 61°C |
| *LY49D* exon 6 | GTGTGATCAGGAGGCTGTG/ TTTCTCAGGGTCGTTAGAGAGT | 586 bp | 61°C |
| *LY49E* exon 6 | GGGTGTGATCAGAAGGCTGT/ CAGGGCCATTAAAGAGAAGGGC | 583 bp | 61°C |
| *LY49F* exon 6 | GGAAGATTATGGGTGTGATCAAT/ TTTGCAGGGTCATTAAAGAGAGG | 601 bp | 61°C |
