# Supplementary material for: Natural Killer Cell Receptor Genes in the Family Equidae: Not only Ly49
Source: PLoS One. 2013 May 28;8(5):e64736. doi: 10.1371/journal.pone.0064736 (PMC3665701; doi:10.1371/journal.pone.0064736)
Supplement: Table S2 — Primer sequences and annealing temperatures used for analysis of KIR -related sequences. (PDF) [file pone.0064736.s005.pdf]

Table S2

Gene/fragment	Length on genomic DNA (cDNA)	Name and sequence of primers	Annealing temperature
KIR3DL	9401 bp (1448 nt)	kir3dl fw: 5’-CTCCTGTTCCGCAGAAGCTA-3’<br>kir3dl rev: 5’-ACAATTGAGCTGTCAAGCTGGGTTT-3’	60°C, 60°C
KIR-ILT	14341 bp (1218 nt)	kir-ilta fw: 5’-TGCGCCATGTCGCCCATGAT-3’<br>kir-ilta rev: 5’-GGGAGCACTCTAGACGTTTTCCTGC-3’	61°C, 61°C
KIRP1	7261 bp	kirp1 fw: 5’-CCTCAGTCTCCTGGGTCTTGGTGA-3’<br>kirp1 rev: 5’-ACCAGGCCTTGGCCAGTGTTG-3’	65°C, 63°C
KIRP2	6254 bp	kirp2 fw: 5’-ACCATGTCCCCCAAATTCCTCAG-3’<br>kirp2 rev: 5’-GATGCTGGACCACCACAAACTTAA-3’	68°C, 63°C
KIR-ILT exon1-exon6	6653 bp	kir-ilta fw: 5’-TGCGCCATGTCGCCCATGAT-3’<br>kir-ilta in6 rev: 5’-GGAAGATGCTAGGGTTGCTG-3’	65°C, 65°C
KIR-ILT fusion	1505 bp	kir-ilta in5 fw: 5’-TGCAGATGCCTTTAACGCAAG-3’<br>kir-ilta in6 rev: 5’-GGAAGATGCTAGGGTTGCTG-3’	60°C
KIR3DL exon3	380 bp	kir3dl in2 fw: 5’-TGGAGGGGAGGAGGTCCCCAT-3’<br>kir3dl in3 rev: 5’-GGGACAGTGAATAGCCACTCTGGA-3’	60°C
KIR3DL exon5	91 bp	kir3dl ex5 fw: 5’-TCCCAGGCCTCTACAAGAAACCTT-3’<br>kir3dl ex5 rev: 5’-TCAGAGCTGCAGAACAAGGTCACA-3’	60°C
KIR3DL exon8-exon9	855 bp	kir3dl in7 fw: 5’-GGCCAGTGCTAGCTGGGCAA-3’<br>kir3dl rev: 5’-ACAATTGAGCTGTCAAGCTGGGTTT-3’	60°C
KIR3DL exon3-exon5	3329 bp	kir3dl ex3 fw: 5’-GTGGTCAGGACAAGCCCTCT-3’<br>kir3dl ex5 rev: 5’-TCAGAGCTGCAGAACAAGGTCACA-3’	60°C - 55°C,55°C
KIR3DL exon5-exon9	5020 bp	kir3dl ex5 fw: 5’-TCCCAGGCCTCTACAAGAAACCTT-3’<br>kir3dl rev: 5’-ACAATTGAGCTGTCAAGCTGGGTTT-3’	50°C, 50°C
