# Supplementary material for: Natural Killer Cell Receptor Genes in the Family Equidae: Not only Ly49
Source: PLoS One. 2013 May 28;8(5):e64736. doi: 10.1371/journal.pone.0064736 (PMC3665701; doi:10.1371/journal.pone.0064736)
Supplement: Table S3 — List of accession numbers of mammalian KIR3DL sequences used for the phylogenetic tree construction. (PDF) [file pone.0064736.s006.pdf]

Table S3

**Name of sequence**	**species**	**accession numbers**
Mus musculus KIR3DL1	house mouse	NP_808417.2; gi 125490384
Mus musculus KIR3DL2	house mouse	NP_808416.1; gi 29244534
Rattus norvegicus	Norway rat	NP_852144.1; gi 31324550
Cricetulus griseus	Chinese hamster	XP_003511503.1; gi 354498804
Cavia porcellus	domestic guinea pig	XP_003465608.1; gi 348559608
Bos taurus KIR3DL1	cattle	NP_852116.1; gi 32189324
Bos taurus KIR3DL2	cattle	NP_001091558.1; gi 148227982
Ovis aries KIR2DL3	sheep	XP_004022885.1; gi 426258575
Ovis aries KIR3DL1-like	sheep	XP_004015947.1; gi 426244270
Ovis aries KIR3DL2-like	sheep	XP_004015946.1; gi 426244268
Loxodonta africana	African savanna elephant	XP_003406656.1; gi 344269639
Bos taurus KIR2DL1	cattle	AAP33624.1; gi 30466080
Sus scrofa KIR2DL1	pig	NP_001106689.1; gi 172073179
Halichoerus grypus	gray seal	ACN73230.1; gi 224980757
Phoca vitulina vitulina	harbor seal	ACN73228.1; gi 224980753
Leptonychotes weddellii	Weddell seal	ACN73224.1; gi 224980745
Zalophus californianus	California sea lion	ACN78956.1; gi 225016054
Ailuropoda melanoleuca	giant panda	XP_002926417.1; gi 301782005
Ceratotherium simum	white rhinoceros	KC412056
Rhinoceros unicornis	greater Indian rhinoceros	KC412055
Equus caballus	horse	KC315949
Equus asinus	donkey	KC315957
Equus asinus somalicus	Somali wild ass	KC315956
Equus hemionus kulan	kulan	KC315959
Equus kiang	kiang	KC315958
Equus grevyi	Grevy's zebra	KC315950
Equus zebra hartmannae	Hartmann's mountain zebra	KC315951
Equus burchellii cunninghami	plains zebra subspecies	KC315955
Equus burchellii chapmani	Chapman's zebra	KC315954
Equus burchellii boehmi	Grant's zebra	KC315953
Equus burchellii antiquorum	Damara zebra	KC315952
